# Data of soil, vegetation and bird species found on double-crested cormorant colonies in the southeastern United States

**DOI:** 10.1016/j.dib.2019.104726

**Published:** 2019-10-29

**Authors:** Leah Moran Veum, Brian S. Dorr, Katie Hanson-Dorr, R.J. Moore, Scott A. Rush

**Affiliations:** a Department of Wildlife, Fisheries, & Aquaculture, Mississippi State University, PO Box 9690, Mississippi State, MS, 39762, USA; b U.S. Department of Agriculture, Wildlife Services, National Wildlife Research Center, Mississippi Field Station, P.O. Box 6099, Mississippi State, MS, 39762, USA; c Tennessee Valley Authority, Land & River Management, Natural Resources, 1010 Reservation Road, MPB 1H-M, Muscle Shoals, AL, 35662, USA

**Keywords:** Waterbird, Southeastern forest, Diversity, Avian, Vegetation, Soil

## Abstract

This data article provides the methods and procedures followed to collect and analyse soil, vegetation and bird data on three different treatment islands in Guntersville Reservoir, Alabama. Samples were collected from randomly selected plot points from islands that were placed into three different treatment types: Colony (currently occupied by Double-crested Cormorants) (*Phalacrocorax auritus*; n = 5), Historic (historically occupied by cormorants and currently abandoned; n = 3) and Reference (never occupied by cormorants; n = 4). We compared vegetation and tree metrics such as structure and diversity, as well as soil chemistry and bird diversity and communities among islands within Guntersville Reservoir. These data document for the first time that we are aware of the long-term effects of soil chemistry changes, vegetation changes, and impacts to avian diversity, in temperate forest ecosystems, by cormorant colonies. All data is associated with the recent article by Veum et al. [1] and provided here as raw data.

**Table:** Specifications Table

Subject	Environmental Science (General)
Specific subject area	Effects of nesting Double-crested Cormorants on soils, and plant and avian communities on insular habitats
Type of data	Table Figure
How data were acquired	Random soil Sampling Random Vegetation plot sampling Random point count survey
Data format	Raw
Parameters for data collection	Soil properties Plant structure and diversity Tree diversity and health Bird diversity and community structure
Description of data collection	Data was collected by physically removing a soil sample from random locations on islands to be analysed in a laboratory while plant and tree species were recorded by observing specimens on site within randomly selected plots or point count locations respectively. Bird species were recorded by observing species or hearing the song associated with that species.
Data source location	Guntersville Reservoir, Guntersville, Alabama, USA
Data accessibility	Data are presented with this article
Related research article	Veum, L. M., Dorr, B. S., Hanson-Dorr, K. C., Moore, R. J., & Rush, S. A. (2019). **Double-crested cormorant colony effects on soil chemistry, vegetation structure and avian diversity.** Forest Ecology and Management, 453, 117588. https://doi.org/10.1016/j.foreco.2019.117588

**Table:** 

**Value of the Data** • Our findings highlight that breeding Double-crested cormorants have long-term impacts to soil, vegetation structure, tree density and health and bird diversity on insular, temperate forest ecosystems in the southeastern U.S [1]. • No previous data exist on impacts of cormorants to bird communities and limited data of effects on soil, water quality, and trees in the southeastern U.S. • This data is useful to scientists conducting future research on the effects of cormorants as well as land managers and regulatory agencies who want to manage and control damage from cormorants and their numbers on breeding grounds • More research is needed to fully understand the indirect effects of cormorant occupancy on bird communities, such as a decrease in avian diversity, which this data can be a basis for • This data provides a baseline reference for future research or further avenues for bird counts and for studies on other species such as small mammals or amphibian communities

## 1 Data

**Fig. 1:**
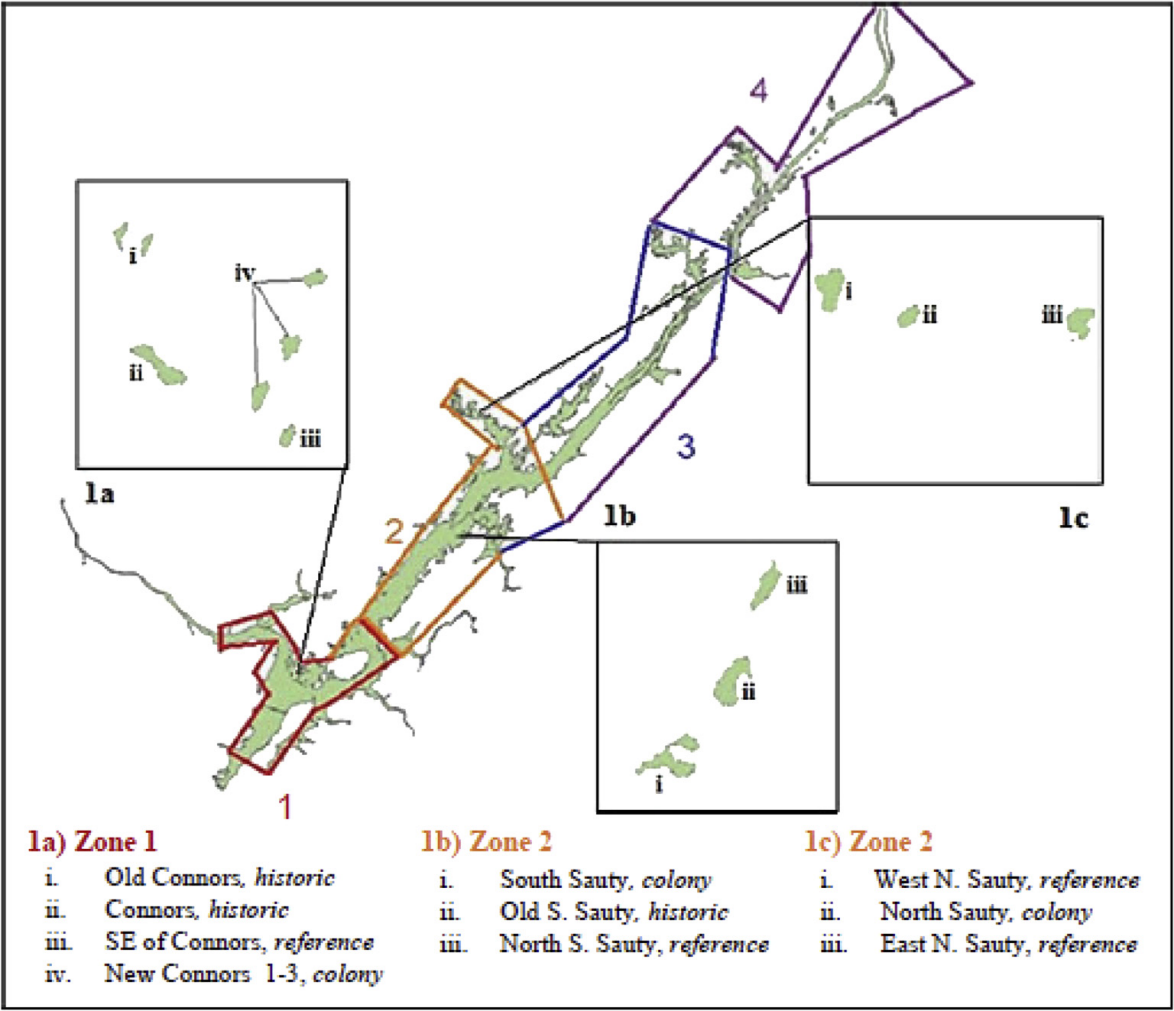
Study area and islands sampled on Guntersville Reservoir, Alabama, June–August 2016 and 2017. Zones 1 and 2, color coded have islands divided into three treatment groups (1a, 1b, 1c): colony (active cormorant colonies), reference (islands with no history of nesting) and historic (abandoned colony islands), for our sampling plots.

The figure provided illustrates the locations of sampled islands on Guntersville Reservoir (Fig. 1). The reservoir is divided into four major zones, with zones 1 and 2 our main focus due to the consistent presence of Double-crested cormorant (*Phalacrocorax auritus*) colonies. These two zones are subdivided into the individual islands sampled and were categorized into three treatment groups: colony, reference and historic. The datasets are tables that contain a comprehensive list of all species of tree, plant and birds recorded, as well as all soils nutrient values extracted from soil samples within islands on Guntersville Reservoir. Soil data (Table 1) is presented as each individual plot point on every island sampled, with column headings for island type and each soil nutrient content extracted. For plants (Table 2) and trees (Table 3) the common and scientific name are given with columns divided into colony, reference and historic with these columns further subdivided into individual islands sampled. The number under each is the total count of each species found on those individual islands. For birds (Table 4), the species name and scientific name are given, with the total count recorded under the columns of colony, historic and reference. Further, a Continental Concern Score was given for each recorded species [12]. All data included are raw values.

**Table 1:** Summary of all soil nutrient concentration extracted from soil samples on plots located in active cormorant colonies (colony), islands with no history of nesting (reference) and abandoned colony islands (historic) on Guntersville Reservoir, Alabama, June–August 2016. Numbers in columns are the value for soil components for every plot point surveyed on all islands.

Island	Island Type	% OM	P	K	Ca	Mg	Zn	S	Na	pH	NH_4_^−^	NO_3_^−^
CON	HISTORIC	4.76	1704	352	2759	56	6.1	685	55	3.9	90.91	175.93
CON	HISTORIC	5.25	788	232	1349	67	5.2	756	17	3.8	38.71	99.82
CON	HISTORIC	2.92	576	100	865	85	3.6	420	15	4.3	15.73	27.31
CON	HISTORIC	3.95	784	160	1004	45	3.1	569	10	4.3	19.06	18.18
CON	HISTORIC	4.55	1036	209	997	40	3.9	655	16	3.6	9.36	84.37
CON	HISTORIC	4.77	932	178	1721	72	5.5	687	17	4.2	20.24	37.78
CON	HISTORIC	4.17	872	157	888	46	3.4	600	11	4.1	14.92	16.73
CON	HISTORIC	3.59	220	169	1604	182	4.6	517	12	4.8	20.84	35.07
CON	HISTORIC	3.68	856	206	1122	59	3.4	530	11	4.1	15	23.83
CON	HISTORIC	3.01	74	64	760	53	1	433	130	4.6	22.33	24.79
CON	HISTORIC	3.05	2002	475	4659	84	10.2	439	78	4.3	75.32	316.45
CON	HISTORIC	4.48	1116	191	2416	51	10.2	645	20	4.4	32.61	70.43
CON	HISTORIC	4.95	1800	168	3165	51	7.5	713	43	4	40.32	141.66
CON	HISTORIC	3.46	2016	149	3473	42	7.9	498	37	4.2	20.14	99.23
CON	HISTORIC	4.39	996	146	1184	54	4	632	20	3.7	10.02	6.25
CON	HISTORIC	4.1	1068	181	1032	41	3	590	10	4.1	10.73	5.5
CON	HISTORIC	2.52	1124	319	850	42	3.3	363	15	3.8	9.33	48.03
CON	HISTORIC	3.07	180	204	1079	102	3.7	442	17	4.1	17.78	53.02
ENS	REFERENCE	4.11	41	127	1487	62	2.7	592	86	5.2	46.59	0.86
ENS	REFERENCE	3.45	9	69	3660	200	2.3	497	40	5.8	8.1	3.74
ENS	REFERENCE	2.71	7	79	3597	152	1.6	390	43	6.4	6.73	0.15
ENS	REFERENCE	1.39	7	29	2169	82	0.6	200	23	6.4	5.31	0.63
ENS	REFERENCE	2.57	23	53	4003	67	1.1	370	44	6.7	7.39	3.3
ENS	REFERENCE	4.88	149	159	4474	186	2.1	703	33	5.8	15.09	6.17
NCON1	COLONY	3.39	584	98	1713	59	3.2	488	56	4.3	17.99	36.34
NCON1	COLONY	4.77	349	153	1922	109	6.1	687	22	4.5	18.16	31.78
NCON1	COLONY	2.71	176	174	666	53	1.4	390	16	4.2	17.65	77.44
NCON1	COLONY	4.07	92	241	1744	194	2.9	586	22	4.7	32.72	25.39
NCON1	COLONY	3.49	53	298	1230	129	4.6	503	18	5.1	6.3	11.51
NCON1	COLONY	4.8	120	244	1669	121	3.9	691	21	4.3	16.11	31.97
NCON1	COLONY	3.39	654	248	1320	57	3.8	488	17	4.1	11.94	73.51
NCON2	COLONY	2.15	88	87	1181	76	3.9	310	20	4.7	9.64	45.8
NCON2	COLONY	3.44	388	99	1560	72	2.4	495	21	4.2	10.81	48.79
NCON2	COLONY	2.85	107	104	1250	158	3.4	410	15	4.6	11.72	49.92
NCON2	COLONY	3.05	488	249	1512	89	4.5	439	28	4.2	18.12	164.25
NCON2	COLONY	2.71	127	151	1089	125	2.1	390	19	4.4	18.94	69.11
NCON2	COLONY	4.47	47	173	1880	281	2.4	644	23	5.1	23.81	34.74
NCON2	COLONY	3.16	48	127	1449	205	2.1	455	14	4.9	15.18	50.74
NCON2	COLONY	2.25	55	213	783	123	2.3	324	11	4.6	6.49	23.9
NCON3	COLONY	2.31	12	89	1814	168	1.7	333	39	6	22.79	13.47
NCON3	COLONY	2.99	180	307	1786	144	4	431	59	4.7	102.81	194.25
NCON3	COLONY	3.76	47	155	2305	270	1.3	541	63	5.4	17.78	17.96
NCON3	COLONY	5.19	386	652	2380	176	3.6	747	56	4.7	29.36	99.7
NCON3	COLONY	3.46	108	147	2496	234	2.3	498	20	5.3	12.24	48.3
NCON3	COLONY	3.3	22	155	2323	244	2.9	475	23	5.4	8.73	27.38
NSAUT	REFERENCE	3.05	97	62	1061	57	0.7	439	31	5.1	8.96	0.94
NSAUT	REFERENCE	5.69	93	132	3181	144	1.9	819	43	5.7	26.5	1.19
NSAUT	REFERENCE	3.41	448	58	2347	104	3.1	491	22	5.3	14.83	14.51
NSAUT	REFERENCE	4.89	750	175	2089	76	6.7	704	33	4.3	47.19	85.79
NSAUT	REFERENCE	4.13	177	80	3479	118	1.9	595	114	5.9	26.15	28.31
NSS	REFERENCE	1.95	71	70	877	79	1.7	281	15	5.3	19.51	1.9
NSS	REFERENCE	2.61	48	87	381	60	1.3	376	16	4.4	22.73	0.88
NSS	REFERENCE	3.49	36	126	1139	171	1.8	503	20	5.1	39.07	0.22
NSS	REFERENCE	2.99	51	125	685	102	1.3	431	24	5	24.03	2.2
NSS	REFERENCE	3.83	39	111	1031	102	1.3	552	16	4.8	28.52	18.19
NSS	REFERENCE	1.79	20	73	277	39	0.7	258	15	4.7	16.87	4.47
NSS	REFERENCE	3.29	34	114	293	48	1.2	474	24	4.3	19.41	0.83
NSS	REFERENCE	2.66	30	74	965	112	1	383	84	4.8	21.59	0.24
OLDC	HISTORIC	3.92	1770	288	5628	97	14.5	564	23	4.9	11.42	23.39
OLDC	HISTORIC	4.88	2944	364	7953	93	16.4	703	26	5.3	17.23	20.7
OLDC	HISTORIC	4.82	1195	335	3950	83	15.6	694	18	5	25.98	47.89
OLDC	HISTORIC	2.88	1372	214	3302	61	11.1	415	15	4.9	7.11	30.57
OLDC	HISTORIC	1.45	1352	206	746	31	3.5	209	12	4.2	10.33	20.26
OLDC	HISTORIC	2.81	782	159	448	47	1.4	405	12	3.9	8	24.1
OSS	HISTORIC	2.48	562	227	1349	78	3.1	357	13	4.7	11.58	28.51
OSS	HISTORIC	3.09	41	96	1916	154	6.8	445	176	5.3	31.1	14.91
OSS	HISTORIC	2.85	502	150	1388	82	4.4	410	13	4.5	9.1	32.46
OSS	HISTORIC	3.91	844	243	1997	134	7.3	563	32	4.4	13.41	33.98
OSS	HISTORIC	4.37	71	76	1895	178	7.1	629	48	5.6	51.78	0.57
OSS	HISTORIC	2.73	266	88	764	47	2.6	393	16	4.5	6.09	20.6
OSS	HISTORIC	2.06	21	64	1467	99	1.6	297	33	5.3	5.54	3
OSS	HISTORIC	2.25	20	75	1970	205	2.8	324	31	5.5	5.68	0.16
OSS	HISTORIC	2.44	126	108	1707	92	2.2	351	16	4.8	9.19	20.22
OSS	HISTORIC	2.38	35	103	2607	205	4.1	343	118	6.1	15.51	7.28
SECON	REFERENCE	3.76	79	74	2078	78	3	541	99	5.6	20.59	10.85
SECON	REFERENCE	2.56	282	172	1701	83	9.6	369	14	5	29.79	37.5
SECON	REFERENCE	4.63	298	171	1736	92	7.4	667	12	4.6	19.27	42.59
SECON	REFERENCE	2.69	160	100	2106	106	15.5	387	16	5.3	6.13	21.25
SECON	REFERENCE	3.04	185	111	1739	109	13	438	14	5.2	18.69	28.89
SSS	COLONY	5.17	1295	240	2952	59	9.5	744	12	4.6	15.68	21.85
SSS	COLONY	4.11	976	366	1223	44	5	592	13	4.3	9.98	31.14
SSS	COLONY	3.53	646	140	1330	84	3.9	508	13	4.5	12.54	23.22
SSS	COLONY	4.42	564	243	1443	100	4.4	636	20	4.1	22.81	58.68
SSS	COLONY	3.34	1195	444	2608	67	9.7	481	24	4.7	16.17	25.98
SSS	COLONY	2.69	1545	448	2618	46	5.5	387	13	4.6	10.2	20.43
SSS	COLONY	4.14	1240	232	1452	38	6.4	596	13	3.7	14.33	45.3
SSS	COLONY	4.79	772	291	3402	130	8	690	44	4.5	53.6	160.29
SSS	COLONY	2.19	716	209	3900	181	5.2	315	101	5.5	11.2	71.31
WNS	REFERENCE	3.69	49	47	615	29	2.2	531	36	4.6	14.74	1.51
WNS	REFERENCE	4.72	29	81	2193	68	1.9	680	45	5.5	41.4	4.57
WNS	REFERENCE	2.43	29	31	423	21	1.3	350	30	4.9	9.11	0.38
WNS	REFERENCE	2.57	34	62	1187	45	2.5	370	27	5.4	42.91	0.73
WNS	REFERENCE	3.15	89	92	1288	83	0.8	454	49	5.3	17.84	0.13
WNS	REFERENCE	1.47	8	38	1020	30	1.1	212	21	5.4	21.01	0.27
WNS	REFERENCE	2.33	16	37	1626	57	1.9	336	22	5.9	28.19	0.69
WNS	REFERENCE	2.12	47	45	779	22	1.7	305	36	5	23.57	0.41

**Table 2:** Summary of all plant species documented on plots located in active cormorant colonies (colony), islands with no history of nesting (reference) and abandoned colony islands (historic) on Guntersville Reservoir, Alabama, June–August 2016. Numbers in table represent total count of each plant species on each individual island, with island abbreviation and treatment group shown in column head.

Common Name	Native Status	Scientific Name	Colony Islands			Reference Islands			Historic Islands		
			NCON1–3	SSS	NSAUT	SECON	NSS	ENS/WNS	OLD C	CON	OSS
Alabama sucklejack	Native	*Berchemia Scandens*			5		18	12			
Alligator weed	Non	*Alternanthera philoxeroides*		149	20						1451
Beefsteak plant	Non	*Perilla frutescens*								53	
Bermuda grass	Non	*Cynodon dactylon*		1							
Black snakeroot	Native	*Sanicula canadensis*							1		
Blackberry bush	Native	*Rubus argutus*	8			23	19	9	11	19	1
Bloodroot	Native	*Sanguinara canadensis*							1		
Canada violet	Native	*Viola canadensis*						179			37
Carolina moonseed	Native	*Cocculus carolina*							4		
Cat greenbriar	Native	*Smilax glauca*	7	4			3			51	9
Chinese lespedeza	Non	*Lespedeza cuneata*			23			6			
Christmas fern	Native	*Polystichum acrostichoides*					5				
Climbing hempvine	Native	*Mikania scandens*						8			
Common wingstem	Native	*Verbesina alternifolia*		224				48			
Creeping burhead	Native	*Echinodorus cordifolius*									24
Devil's darning needle	Native	*Clematis virginiana*								5	
Ebony spleenwort	Native	*Asplenium platyneuron*									2
Elderberry	Native	*Sambucus nigra*	10	49				2	57	3	6
Goldenrod	Native	*Solidago canadensis*									76
Grass	Native	*Poaceae Family*						35			
Gray's sedge	Native	*Carex grayi*						10			21
Gr. Marsh St. John's wart	Native	*Hypericum walteri*						53			
Hazel alder	Native	*Alnus serrulata*	3								
Horseweed	Native	*Conyza canadensis*			54						
Indian strawberry	Non	*Duchesnea indica*		1						2	15
Japanese honeysuckle	Non	*Lonicera japonica*	17			12	27	3	171	23	15
Jewelweed	Native	*Impatiens capensis*	6	5	5	1					76
Lamb's quarter	Non	*Chenopodium album*		1							
Lanceleaf greenbriar	Native	*Smilax smallii*	31		10	20	23	6	1	3	12
Late flowering boneset	Native	*Eupatorium serotinum*		11					6		
Lizard's tail	Native	*Saururus cernuus*	4		35			155			
Morning glory	Non	*Ipomoea purpurea*	39		1		38				6
Muscadine grape	Native	*Vitis rotundifolia*	52	9	10	6	27	1		1	18
Oatgrass	Native	*Danthonia* sp.			1						4
Partridge berry	Native	*Mitchella repens*		6							
Passion flower	Native	*Passiflora incarnata*				7	2	1			
Plantain	Non	*Plantago* sp.		1							15
Poison ivy	Native	*Toxicodendron radicans*	17	1	216		123	60	6		17
Pokeweed	Native	*Phytolaca americana*	285	468	30	1			906	1221	118
Potato bean	Native	*Apios americana*								3	
Privet	Non	*Ligustrum* sp.	207	7			16			1	46
Roundleaf greenbrier	Native	*Smilax rotundifolia*	24	1	1			8		3	6
Saw greenbriar	Native	*Smilax bona-nox*	21								
Sawgrass	Non	*Cladium* sp.									1
Smartweed	Native	*Polygonum* sp.	1180			1		1	8		
Smooth ticktrefoil	Native	*Desmodium laevigatum*			9						
St. Andrew's cross	Native	*Hypericum hypercoides*			2						
Star cucumber	Native	*Sicyos angulatus*							2		
Stinging nettle	Non	*Urtica dioca*					44	44			73
Strawberry bush	Native	*Euonymus americanus*			1						
Swamp dogwood	Native	*Cornus racemosa*						3			
Leather flower	Native	*Clematis crispa*	2						1	5	1
Switch cane	Native	*Arundinaria gigantea*								11	
Threeawn grass	Native	*Aristida* sp.					10				
Trumpet creeper	Native	*Campsis radicans*	10		3		10	5	3	22	4
Virginia creeper	Native	*Parthenocissus quinquefolia*	10		3		31				6
Virginia dayflower	Native	*Commelina virginica*	89		11		41	287		43	682
Water pennywort	Native	*Hydrocotyle* sp.						53			85
Wild cotton	Native	*Hibiscus moscheotos*	4								
Wild grape	Native	*Vitis aestivalis*				1					1
Wild Taro	Non	*Colocasia esculenta*	19								
Woodland lettuce	Native	*Lactuca floridana*	9	52	56	18			21		26
Yam-leaved clematis	Non	*Clematis terniflora*								1	
Yellow woodsorrel	Native	*Oxalis Stricta*	9

**Table 3:** Summary of all tree species documented on plots located on islands with active cormorant colonies (colony), islands with no history of nesting (reference) and abandoned colony islands (historic) on Guntersville Reservoir, Alabama, June–August 2016. Values for each tree species are total count by species found on each individual island, where island abbreviation and treatment group is above each column.

Common Name	Scientific Name	Colony Islands			Reference Islands			Historic Islands		
		NCON1–3	SSS	NSAUT	SECON	NSS	ENS/WNS	OLDC	CON	OSS
American elm	*Ulmus americana*	1					6			
American hornbeam	*Carpinus caroliniana*					9	124			
American sycamore	*Platanus occidentalis*					1				
Bald cypress	*Taxodium distichum*		2							6
Black cherry	*Prunus serotina*	12		11	2	72	3		6	
Black locust	*Robinia pseudoacacia*	29			15	34	15	110	19	7
Black oak	*Quercus velutina*	23		5		94	63		2	
Black gum	*Nyssa sylvatica*						3			
Boxelder	*Acer negundo*	5			3	1			1	11
Buttonbush	*Cephalanthus occidentalis*						2			
Carolina buckthorn	*Rhamnus caroliniana*	35		9	4	1				
China berry	*Melia azedarach*	2								
Common persimmon	*Diospyros virginiana*	12		31		47	74		3	3
Devil's walking stick	*Aralia spinosa*	2				2		4	140	
Eastern redbud	*Cercis canadensis*	4		2		10	2			
Eastern red cedar	*Junipera virginiana*			6		20	4			
Flowering dogwood	*Cornus florida*					19	10		1	
Green ash	*Fraxinus pennsylvanica*						1			
Hackberry	*Celtis occidentalis*	7		3	8	2		9	3	1
Honey locust	*Gleditsia triacanthos*	5								
Loblolly pine	*Pinus taeda*	11	2		4	2			23	2
Mimosa	*Albizia julibrissin*						2			
Mockernut hickory	*Carya tomentosa*	1								
Pawpaw	*Asimina triloba*						16			
Post oak	Quercus stellata								1	
Red buckeye	*Aesculus pavia*			20			3			
Red maple	*Acer rubrum*	16	1	22	1	14	41		11	2
Red mulberry	*Morus rubra*				19	1			6	
Sassafras	*Sassafras albidum*	1		2	1	11			102	
Silver maple	*Acer saccharinum*	7	2	2	2			2	2	1
Swamp chestnut oak	*Quercus michauxii*			12		7	5			
Sweet gum	*Liquidambar styraciflua*	2		17		23	114			
Tulip poplar	*Liriodendron tulipifera*	4	5	1	5	20	19		14	4
Water oak	*Quercus nigra*			20		54	96			
White ash	*Fraxinus americana*	3					1			
Willow oak	*Quercus phellos*						11			
Winged elm	*Ulmus alata*					1				
Winged sumac	*Rhus copallinum*						3			
Oak sp.	*Quercus* sp.		1							
Unknown genus						1		3	3

**Table 4:** Summary of all avian species documented on active cormorant colonies (colony), islands with no history of nesting (reference) and abandoned colony islands (historic) on Guntersville Reservoir, Alabama, June–August 2017.

Species	Scientific Name	Colony	Historic	Reference	CCS
American Crow	*Corvus brachyrhynchos*	4	5	3	7
Barn Swallow	*Hirundo rustica*			1	8
Belted Kingfisher	*Megaceryle alcyon*	2	2		10
Blue Jay	*Cyanocitta cristata*		1	2	8
Blue-gray Gnatcatcher	*Polioptila caerulea*		1	5	7
Blue-winged warbler	*Vermivora cyanoptera*	4			13
Brown-headed Cowbird	*Molothrus ater*	4		1	7
Brown-headed Nuthatch	*Sitta pusilla*	1		3	13
Canada Goose	*Branta canadensis*		2	1	6
Carolina Chickadee	*Poecile carolinensis*	10	8	17	9
Carolina Wren	*Thryothorus ludovicianus*	25	36	26	7
Common Grackle	*Quiscalus quiscula*	36	28	15	9
Common Yellowthroat	*Geothlypis trichas*	2	1		9
Downy Woodpecker	*Picoides pubescens*	1		1	7
Eastern Kingbird	*Tyrannus turannus*	9	9	15	11
Eastern Phoebe	*Sayornis phoebe*		2	3	8
Eastern Towhee	*Pipilo erythrophthalmus*	10	10	25	11
Eastern Wood-peewee	*Contopus virens*	1	1	17	10
European Starling	*Sturnus vulgaris*	1	4	3	7
Fish Crow	*Corvus ossifragus*	2	8	1	10
Great-blue Heron	*Ardea herodias*	2	4	4	8
Hairy Woodpecker	*Leuconotopicus villosus*			1	6
House Finch	*Haemorhous mexicanus*			1	6
House Sparrow	*Passer domesticus*			1	8
House Wren	*Troglodytes aedon*			1	5
Indigo Bunting	*Passerina cyanea*			1	9
Mourning Dove	*Zenaida macroura*			5	6
Northern Cardinal	*Cardinalis*	66	63	54	5
Northern Flicker	*Colaptes auratus*		1	3	9
Northern Mockingbird	*Mimus polyglottus*		2	1	8
Orchard Oriole	*Icterus spurius*	3	4		10
Osprey	*Pandion haliaetus*	8	11	15	7
Pileated Woodpecker	*Hylatomus pileatus*		2	2	7
Pine Warbler	*Setophaga pinus*	6	1	3	7
Prairie Warbler	*Setophaga discolor*	1			14
Prothonotary Warbler	*Protonotaria citrea*	1	2		14
Purple Martin	*Progne subis*		7		10
Red-bellied Woodpecker	*Melanerpe carolinus*	1	4	5	7
Red-eyed Vireo	*Vireo olivaceus*		2	1	6
Red-headed Woodpecker	*Setophaga pinus*	3	13	2	13
Red-winged Blackbird	*Agelaius phoeniceus*	3	4	8	8
Ruby-throated Hummingbird	*Archilochus colubris*	2	2		8
Tufted Titmouse	*Baeolophus bicolor*	4	8	16	7
White-breasted Nuthatch	*Sitta carolinensis*			2	6
White-eyed Vireo	*Vireo griseus*			2	8
Yellow-bellied Sapsucker	*Sphyrapicus varius*			1	7
Yellow-billed Cuckoo	*Coccyzus americanus*		7	4	12
Yellow-breasted Chat	*Icteria virens*			1	10
Yellow-throated Warbler	*Setophaga dominica*	4		3	10

## 2 Experimental design, materials, and methods

Twelve islands were sampled in Guntersville Reservoir, with these islands divided into three treatment groups: colony (islands colonized by breeding cormorants), reference (islands with no cormorant occupancy) and historic (islands that were colonized by cormorants but subsequently abandoned). Five islands were categorized as colony, four as reference and three as historic islands. Colony islands included New Connors 1, New Connors 2, New Connors 3, South Sauty and North Sauty. Reference islands were selected based on proximity and a similar area to colony islands. Reference islands included SE Connors, North South Sauty, West North Sauty and East North Sauty. Historic islands included Old Connors, Connors and Old South Sauty.

A stratified random sampling approach was used to obtain locations on islands to collect all data. This sampling design was based on island size where the density of samples per unit of effort was constant across islands of differing size. We sampled each island multiple times (multiple plot points on islands) and therefore made whole island, not plot level, inferences. Almost all sample locations were determined from plots referenced in Lafferty et al. [2]. Due to erosion or inaccessibility, some plots needed to be replaced which was accomplished by overlaying a 10 meter × 10 meter (m) grid over the islands and selecting plots by proportionally sampling 20% of the 10m^2^ grid on each island [2]. This grid was created using orthoquad imagery of Guntersville Reservoir and ArcMap v.10.1 (ESRI, 2012). Plot center was determined by recording the latitude and longitude at the center of the plot.

### 2.1 Soil

Soil sampling was done by placing a 1m^2^ quadrat made of PVC pipe at plot center. The surface detritus and litter layers were brushed away and the soil sample was taken from the center of the 1m^2^ plot to a depth of 22 centimeters (cm) using a soil auger (9 cm diameter) and hand trowel. Once collected, soil was homogenized and kept cool and dry until all soil collections were completed. For lab processing, nutrient concentrations (kg/ha) and base saturation were extracted from each sample which were used to determine percent concentrations for the following soil characteristics: percent organic material (%OM), pH, phosphorus (P), potassium (K), calcium (Ca), magnesium (Mg), zinc (Zn), sulfur (S), sodium (Na), hydrogen (H), nitrate (NO_3_^−^) and ammonium (NH_4_^−^). These nutrients were selected because of their importance in plant physiology and circulation and to their correlation with excess cormorant fecal deposits [1,3,4].

### 2.2 Community diversity

Habitat characteristics were measured at the same plot points for soil sampling. The same 1m^2^ quadrat was placed at plot center to measure percent plant cover, plant density, and plant diversity following procedures developed by Ayers et al. [5]. A digital image was taken of the plot before any further disturbance so that percent cover could be calculated. This was completed by uploading each image onto a computer and overlaying a grid comprised of 100 equal squares over the image [5]. Each box was recorded as covered (≥50% of the box covered by live vegetation) or not covered (≤50% covered by live vegetation). Once all 100 boxes were recorded for an image, the number of covered boxes indicated live plant cover for that plot. Plant diversity was recorded by identifying all species in a plot and plant density was recorded by counting each individual of that species in each plot. Any plants that could not be identified were given a unique number and pressed for future identification with the density of these unknown species still counted and recorded.

Canopy cover was measured using a spherical densiometer [6] in each cardinal direction at 5 m from plot center. Percent canopy recorded in each direction was used to calculate average canopy cover for each plot. A Nudd's board [7] was used to measure vegetation density of midstory heights in two random, cardinal directions at 15 m from plot center. The proportion of each 0.5 m (0–2 m) interval covered by vegetation was recorded as a categorical value between 1 and 5 where: (1) 0–20%, (2) 21–40%, (3) 41–60%, (4) 61–80% and (5) 81–100% of vegetation cover [7]. Coverage values were averaged to obtain a single midstory value for each plot.

At all plot points, all tree species in a 10 m radius from plot center were identified, with individual trees that had a diameter at breast height (DBH) of over 8 cm given a unique number, and DBH and vigor class recorded. The vigor class scale was a metric for how healthy a tree was on a scale of 1–5 where: (1) No decay, 100% healthy; (2) Mostly healthy, < 25% decay; (3) Not healthy and/or dying, > 50% decay; (4) Newly dead, 100% decay; and (5) Old snag [2]. Trees less than 8 cm were identified to species and a count of each species recorded. Each plant and tree species was designated as native or non-native using data from the USDA Plant Database [8].

Point count surveys of bird species were conducted on all islands. A point count records all birds heard or seen at a fixed spot for a fixed amount of time [9,10]. A bulls’ eye sheet was used during the survey to document the species of bird, the distance from the observer, relative direction, and time detected [9,10]. Points were not randomly selected due to the small size of the islands and the recommendation that points be at least 200 m away from each other [10]. Because all islands, except Connors Island, were less than 200 m in size, one point was selected as close to the center of each island as possible for an even, whole island recording. For Connors Island, two points were selected that were over 200 m apart from each other and centered at opposite ends of the island. Once plots were determined, point counts were started and repeated six times at each location, with one week between survey times.

Islands were split by cormorant, colony complex groups (Connors, South Sauty and North Sauty; Fig. 1) and split between two recording teams. Connors Island complex had six islands total, therefore colony complex groups where split into two, Connors Islands and North and South Sauty Islands (Fig. 1). The Connors Island complex was further subdivided by reference and historic (e.g., SE Connors, Connors and Old Connors Islands) and current colony islands (e.g., New Connors 1, 2 & 3), with recording teams alternating between the two every trip. South and North Sauty complexes had three islands each, therefore one group collected data at South Sauty complex and the other team at the North Sauty complex, alternating every visit (Fig. 1). Before point counts began, we selected colony island complexes at random (‘Connors’ or ‘Sautys’) and then islands within complexes at random so no island was recorded at the same time of day for a visit. For the first survey, a complex was selected by flipping a coin. Successively, teams alternated the starting complex for each visit. Two days were designated for data collection, ‘Connors’ complex one day and ‘Sautys’ for another, unless weather delayed field work.

Point count surveys began at dawn, which was established by using a weather application. Once at the point, the observer waited 5 minutes before starting to minimize effects of disturbance from arrival. After the waiting period, a 10 minute point count survey began, documenting all birds heard and seen during the 10 minutes. All flyover birds were recorded on the data sheet as well as weather characteristics (wind, cloud cover, rain). A conservation concern score was given to all species found on the plot points. This score was obtained from Partners in Flight Avian Conservation Assessment Database (PIF; Panjabi et al. [11]).
